# Transmission of Disease by Means of Books

**Published:** 1911-12

**Authors:** Wm. R. Reinick

**Affiliations:** 1709 Wallace Street, Philadelphia, Pa.


### CORRESPONDENCE

---

### Transmission of Disease by Means of Books

THE undersigned is preparing a paper upon "Books as a source of disease" to be read before the next "International Congress of Hygiene," and in order to obtain data, respectfully requests the readers of this note to send him an account of any cases, the source of which has been traced to books or papers, or in which the evidence seemed to make books or papers the offender. He would also further request information where illness or even death has been caused by the poisons used in book-making.

All the information possible is wanted to present as complete a paper as possible. As in the case of insects which we now know to be "carriers of disease," it is first necessary to collect the scattered evidence in order to show that there is real danger in books; and this will compel better care to be taken of libraries and books and improve the health of mankind.

WM. R. REINICK,
1709 Wallace Street,
Philadelphia, Pa.

